# Hemicolectomy Does Not Provide Survival Benefit for Right-Sided Mucinous Colon Adenocarcinoma

**DOI:** 10.3389/fonc.2020.608836

**Published:** 2021-02-01

**Authors:** Jia Huang, Qiulin Huang, Rong Tang, Guodong Chen, Yiwei Zhang, Rongfang He, Xuyu Zu, Kai Fu, Xiuda Peng, Shuai Xiao

**Affiliations:** ^1^ Institute of Clinical Medicine, the First Affiliated Hospital, University of South China, Hengyang, China; ^2^ Hengyang Medical College, University of South China, Hengyang, China; ^3^ Department of Gastrointestinal Surgery, the First Affiliated Hospital, University of South China, Hengyang, China; ^4^ Department of Surgery, the First Affiliated Hospital, University of South China, Hengyang, China; ^5^ Department of Pathology, the First Affiliated Hospital, Hengyang, China; ^6^ Institute of Molecular Precision Medicine and Hunan Key Laboratory of Molecular Precision Medicine, Xiangya Hospital, Central South University, Changsha, China; ^7^ Department of Surgery, the Second Affiliated Hospital, University of South China, Hengyang, China

**Keywords:** right-sided colon cancer, mucinous adenocarcinoma, partial colectomy, right hemicolectomy, survival

## Abstract

**Background:**

The extent of bowel resection is widely debated in colon cancer surgery. Right hemicolectomy (RHC) and partial colectomy (PC) are the most common operation options for right-sided colon cancer (RCC). However, there are still no treatment guidelines or published studies to guide surgical options for mucinous adenocarcinoma (MAC) of RCC.

**Methods:**

Patients with MAC and non-specific adenocarcinoma (AC) of RCC who underwent RHC and PC from 2010 to 2015 in the Surveillance, Epidemiology, and End Results (SEER) database were retrieved. The general characteristics and survival were compared and analyzed.

**Results:**

A total of 27,910 RCC patients were enrolled in this study, among them 3,413 were MAC. The results showed that race, carcinoembryonic antigen (CEA) level, perineural invasion (PNI), tumor size, tumor location, TNM stage, liver metastasis, chemotherapy were significantly different between MAC and AC groups. The MAC group had similar dissected lymph nodes, but more positive lymph nodes than the AC group. The overall survival (OS) of the MAC group was poorer than that of the AC group, but cancer-specific survival (CSS) was similar between the two groups. The RHC subgroup of the MAC group had more patients of age ≤60 years, larger tumor size, cecum/ascending colon location and dissected lymph nodes than the PC subgroup, but similar positive lymph nodes, perioperative mortality, OS and CSS as the PC subgroup. Moreover, the univariate and multivariable analyses for the survival of RCC patients with MAC showed that RHC might not be a superior predictor for OS and CSS compared with PC.

**Conclusions:**

RHC could not dissect more positive lymph nodes or provide long-term survival benefits for RCC patients with MAC compared with PC. This study could provide some evidence for surgery treatment selection for MAC of RCC, which has important clinical value in individual management of colon cancer patients.

## Introduction

Colorectal cancer (CRC) is the third most common cancer and the second leading cause of cancer death in the world (1). Surgical resection is the predominant and standard therapy option for CRC (2, 3). Right-sided colon cancer (RCC) occurs in the cecum ascending colon, hepatic flexure and/or transverse colon, and its long-term survival after curative surgery is worse than that of left-sided colon cancer (4). Mucinous adenocarcinoma (MAC) is the second most common histopathological type of CRC, which more often occurs in the RCC (1, 2, 5). MAC is different from non-specific adenocarcinoma (AC) of CRC in the oncologic behavior, genomics, and clinicopathological characteristic (6), and it has a worse prognosis than AC of CRC based on previous studies (7, 8).

The extent of bowel resection for CRC is widely debated, especially in RCC (9). Right hemicolectomy (RHC) and partial colectomy (PC) are the most common operation options for RCC (10, 11). The main difference among the surgery options is the range of bowel resection, and all of them would perform adequate lymph node dissection for RCC treatment. PC means colectomy with longitudinal resection margins are within 10 cm beyond the tumor because lymph node metastases are rarely greater than 10 cm (12). RHC means all of right colon, and a portion of transverse was removed. However, most surgeons generally intend to choose RHC instead of PC for the following potential subjective reasons: first, extensive resection would remove more lymph nodes and supply vessels of the tumor; second, extensive resection might provide better survival but similar complications than PC; the third possible reason is that the operative technique of RHC is not difficult to master, and the process of RHC is more easily and widely publicized for surgeons with pride (3, 4, 10, 13).

However, there is no high-level evidence to show that RHC has any specific benefits for the long-term survival of RCC, as well as MAC of RCC. Instead, it might increase the perioperative complications and mortality as well as reduce the quality of life for RCC patients (14). In addition, studies showing that the number of lymph node metastases of MAC is relatively fewer than that of non-MAC (15). Moreover, there are no study or treatment guidelines to specifically recommend surgical options for RCC according to the histopathological subtype. These thought-provoking studies caused the rethinking of the value of RHC for MAC of RCC.

In this study, we performed a retrospective population-based investigation to explore whether RHC is justified for MAC of RCC based on overall survival (OS) and cancer-specific survival (CSS).

## Methods

### Data Source

We collected data from the SEER cancer registry, which covers approximately 28% of the United States (US) population. SEER is an open and reliable database that provides demographic, epidemiological, tumor location and size and survival data. We required cases from 18 SEER registries in the anonymous data and obtained permission to download the data from the SEER database, which did not require informed patient consent.

### Patient Selection

**Figure 1:**
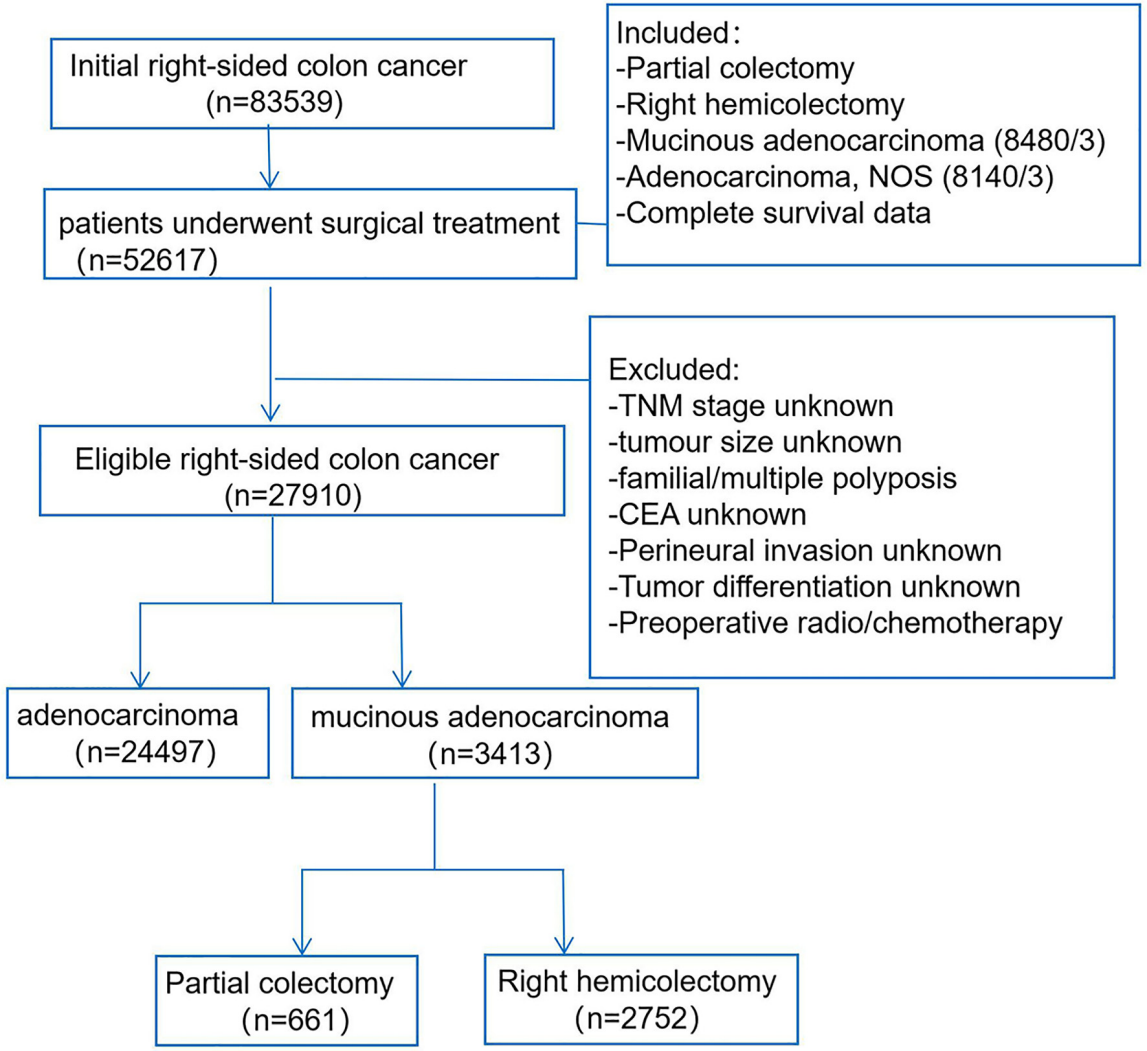
Patient selection flowchart.

We accessed the SEER database by the SEER software (SEER*Stat 8.3.6), and patients who were diagnosed with RCC from 2010 to 2015 were enrolled (**Figure 1**). The study included RCC patients according to the following criteria: 1) treatment with surgical resection, surgical types including RHC (code 40) or PC (code 30); 2) the primary tumor sites were categorized as cecum, ascending colon, hepatic flexure, and transverse colon; 3) the patients had positive histology, and the morphology ICD-0–3 codes of MAC were limited to mucinous adenocarcinoma (8,480/3), the control AC group codes were limited to adenocarcinoma NOS (8,140/3); and 4) exact and complete follow-up information was included. The exclusion criteria: the stage, tumor size, carcinoembryonic antigen (CEA), perineural invasion (PNI), tumor differentiation were unknown, and patients who accepted preoperative chemoradiotherapy were also excluded. Furthermore, the other baseline data were extracted for all patients in the SEER database: race, age, sex, tumor location, tumor number, distant metastasis, perioperative mortality, and postoperative chemotherapy.

### Statistical Analysis

Descriptive statistics of patient characteristics were summarized, and we compared differences in baseline characteristics between the MAC and AC groups in the RCC patients, as well as between the PC and RHC subgroups in the MAC of RCC patients. Continuous data were compared using the one-way ANOVA test, and categorical variables were compared using the chi-square test. For each patient, the survival outcomes were analyzed: 1) overall survival (OS), which was represented as the time from the date of diagnosis to death from any cause; 2) cancer-specific survival (CSS), which was defined as the time from the date of diagnosis until cancer metastasis or recurrence, cancer-associated death and the end of follow-up. Both OS and CSS were estimated using Kaplan–Meier survival curves, and the log-rank test was used to compare the differences among groups. The prognostic factors associated with OS and CSS were analyzed by univariate and multivariable Cox proportional regression. All statistical analyses were performed with the software package SPSS version 22.0 (SPSS Inc., Chicago, IL, USA), and a P value <0.05 was considered statistically significant.

## Results

### General Characteristics and Survival of Right-Sided Colon Cancer Patients With Mucinous Adenocarcinoma

The baseline demographic, clinicopathological, and surgery features of RCC patients were analyzed and compared in **Table 1**, including 3,413 (16.2%) patients with MAC and 24,497 (83.8%) patients with AC. The results showed that the MAC group had a higher proportion of white patients, elevated CEA level, tumor size over 5 cm, tumor location at the cecum, positive lymph nodes, liver metastases, postoperative chemotherapy, and advanced TNM stage than the AC group (P < 0.05). However, the surgery type, dissected lymph nodes, perioperative mortality were similar between the two groups (both P > 0.05).

**Table 1:** The baseline demographic, clinicopathological and surgery features of mucinous adenocarcinoma (MAC) and non-specific adenocarcinoma (AC) of right colon cancer (RCC) patients.

Variables	MAC (3,413)	AC (24,497)	P value
**Race**			
White	2,839(83.2%)	19,457(79.4%)	
Black	356(10.4%)	3,054(12.5%)	
Others	218(6.4%)	1,986(8.1%)	<0.001
**Age (years)**			
≤60	795(23.3%)	5572(22.7%)	
>60	2618(76.7%)	18,925(77.3%)	0.475
**Sex**			
Female	1,869(54.8%)	13,050(53.3%)	
Male	1,544(45.2%)	11,447(46.7%)	0.102
**Surgery type**			
RHC	2,752(80.6%)	19,583(79.9%)	
PC	661(19.4%)	4,914(20.1%)	0.343
**CEA**			
Normal	1,773(50.7%)	14,186(57.9%)	
Elevated	1,640(48.1%)	10,311(42.1%)	<0.001
**PNI**			
Absent	3,100(90.8%)	21,398(87.3%)	
Present	313(9.2%)	3,099(12.7%)	<0.001
**Size (cm)**			
≤5	1,485(43.5%)	14,776(60.3%)	
>5	1,928(56.5%)	9,721(39.7%)	<0.001
**Tumor number**			
Solitary	2,396(70.2%)	17,583(71.8%)	
Multiple	1,017(29.8%)	6,914(28.2%)	0.056
**Location**			
Cecum	1,417(41.5%)	9,373(38.3%)	
Ascending Colon	1,185(34.7%)	8,688(35.5%)	
Hepatic Flexure	274(8.0%)	2,068(8.4%)	
Transverse Colon	537(15.7%)	4,368(17.8%)	0.001
**Differentiation**			
Grade I/II	2,603(76.3%)	18,372(75.0%)	
Grade III/IV	810(23.7%)	6,125(25.0%)	0.108
**Stage (TNM 7ed)**			
I	381(11.2%)	3,954(16.1%)	
II	1,364(40.0%)	8,961(36.6%)	
III	1,185(34.7%)	8,005(32.7%)	
IV	483(14.2%)	3,577(14.6%)	<0.001
**Bone metastases**			
No	3,386(99.2%)	24,324(99.3%)	
Yes	8(0.2%)	73(0.3%)	
Unknown	19(0.6%)	100(0.4%)	0.374
**Brain metastases**			
No	3,396(99.5%)	24,372(99.5%)	
Yes	1(0.0%)	16(0.1%)	
Unknown	16(0.5%)	109(0.4%)	0.713
**Liver metastases**			
No	3,134(91.8%)	21,839(89.1%)	
Yes	273(8.0%)	2,593 (10.6%)	
Unknown	6(0.2%)	65 (0.3%)	<0.001
**Lung metastases**			
No	3,338(97.8%)	23,841(97.3%)	
Yes	57(1.7%)	539(2.2%)	
Unknown	18(0.5%)	117(0.5%)	0.124
**Perioperative mortality**			
Yes	82(2.4%)	680(2.8%)	
No	3,331(97.6%)	23,817(97.2%)	0.210
**Postoperative chemotherapy**			
Yes	1,278(37.4%)	8,523(34.8%)	
No/Unknown	2,135(62.6%)	15,974(65.2%)	0.002
**Dissected lymph nodes**	21.08 ± 10.120	20.72 ± 9.996	0.059
**Positive lymph nodes**	2.41 ± 4.690	2.01 ± 3.923	<0.001

CEA, carcinoembryonic antigen; PNI, perineural invasion; TNM, tumor-node-metastasis; RHC, right hemicolectomy; PC, partial colectomy.

**Figure 2:**
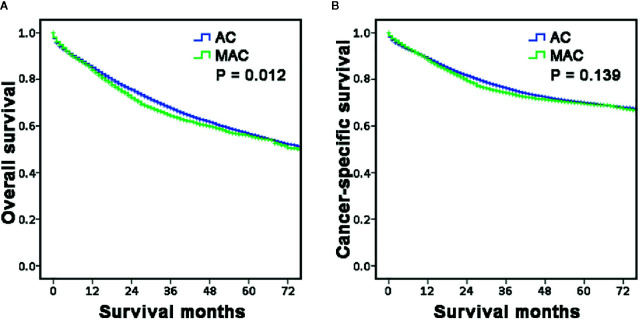
Long-term survival of RCC according to histopathology type. **(A, B)** The survival curves showed that the MAC of RCC group had worse OS **(A)** but similar CSS **(B)** with the AC of RCC group. RCC, right-sided colon carcinoma; OS, overall survival; CSS, cancer-specific survival; MAC, mucinous adenocarcinoma; AC, non-specific adenocarcinoma.

Then, the survival between the two groups was also compared using Kaplan–Meier curves. The results showed that OS of the MAC group was poorer than that of the AC group (P = 0.012, **Figure 2A**), but the CSS was comparable between the two groups (P = 0.139, **Figure 2B**). These results included the general characteristics and long-term survival of MAC of RCC, which indicated that MAC was different from AC.

### Patient Characteristics of Right-Sided Colon Cancer With Mucinous Adenocarcinoma According To Surgery Type

Then, we explored the characteristics of RCC with MAC according to surgery type. The results are shown in **Table 2**, including 661 patients who underwent PC and 2,752 patients who underwent RHC. The RHC group of MAC had a higher proportion of age ≤60 years, tumor size >5 cm and tumor location at cecum/ascending colon than the PC group (P < 0.05, respectively). There were no significant differences for race, sex, CEA, PNI, tumor differentiation, stage, or postoperative chemotherapy (P > 0.05, respectively). More interestingly, the number of dissected lymph nodes in the RHC group was more than that in the PC group (P < 0.001), but the number of positive lymph nodes between the two groups was not significantly different (P = 0.130). What’s more, the perioperative mortality is similar between the two groups (P = 0.272). These results showed the different characteristics of MAC patients who underwent PC or RHC, which indicated the surgeon selection preferences and no benefit of positive lymph node removal.

**Table 2:** The demographic and clinicopathological features of MAC of RCC patients according to surgery type.

Variables	PC (661)	RHC (2752)	P value
**Race**			
White	550(83.2%)	2,289(83.2%)	
Black	70(10.6%)	286(10.4%)	
Others	41(6.2%)	177(6.4%)	0.969
**Age (60 years)**			
≤ 60	131(19.8%)	664(24.1%)	
>60	530(80.2%)	2,088(75.9%)	0.019
**Sex**			
Female	374(56.6%)	1,495(54.3%)	
Male	287(43.4%)	1,257(45.7%)	0.295
**CEA**			
Normal	361(54.6%)	1,412(51.3%)	
Elevated	300(45.4%)	1,340(48.7%)	0.127
**PNI**			
Absent	600(90.8%)	2,500(90.8%)	
Present	61(9.2%)	252(9.2%)	0.954
**Size (cm)**			
≤5	325(49.2%)	1,160(42.2%)	
>5	336(50.8%)	1,592(57.8%)	0.001
**Tumor number**			
Solitary	453(68.5%)	1943(70.6%)	
Multiple	208(31.5%)	809(29.4%)	0.296
**Location**			
Cecum	231(34.9%)	1,186(43.1%)	
Ascending Colon	172(26.0%)	1,013(36.8%)	
Hepatic Flexure	40(6.1%)	234(8.5%)	
Transverse Colon	218(33.0%)	319(11.6%)	<0.001
**Differentiation**			
Grade I/Grade II	502(75.9%)	2,101(76.3%)	
Grade III/Grade IV	159(24.1%)	651(23.7%)	0.829
**Stage(TNM 7ed)**			
I	90(13.6%)	291(10.6%)	
II	268(40.5%)	1,096(39.8%)	
III	212(32.1%)	973(35.4%)	
IV	91(13.8%)	392(14.2%)	0.101
**Perioperative mortality**			
Yes	12(1.8%)	70(2.5%)	
No	649(98.2%)	2,682(97.5%)	0.272
**Postoperative chemotherapy**			
Yes	228(34.5%)	1,050(38.2%)	
No/Unknown	433(65.5%)	1,702(61.8%)	0.081
**Dissected lymph nodes**	18.96 ± 9.477	21.58 ± 10.206	<0.001
**Positive lymph nodes**	2.160 ± 4.479	2.47 ± 4.737	0.130

### Risk Factors for Long-Term Survival of of Right-Sided Colon Cancer With Mucinous Adenocarcinoma

Next, the risk factors for survival of RCC with MAC who accept PC or RHC were analyzed by univariate or multivariable analyses (**Tables 3**, **4**). The results showed that age, CEA level, PNI, tumor size, tumor number, differentiation, and TNM stage were significant prognostic factors for OS in MAC of RCC in univariate analyses (P < 0.05). The association remained significant in multivariable analyses that excluded tumor size (P = 0.863), but included postoperative chemotherapy (P < 0.001). However, tumor location and surgery type were not significant for OS (both P > 0.05, **Table 3**).

**Table 3:** Univariate and multivariable analysis of factors associated with overall survival of MAC of RCC.

Variable	Univariate		Multivariable	
	RR(95%CI)	*P*	RR(95%CI)	*P*
**Race**				
White	1	0.215		
Black	0.970(0.809–1.164)			
Others	0.800(0.623–1.028)			
**Age (years)**				
≤60	1	<0.001	1	<0.001
>60	1.672(1.443–1.938)		1.783(1.525–2.084)	
**Sex**				
Female	1	0.707		
Male	0.979(0.876–1.094)			
**CEA**				
Normal	1	<0.001	1	<0.001
Elevated	1.927(1.722–2.157)		1.467(1.300–1.656)	
**PNI**				
Absent	1	<0.001		0.001
Present	2.034(1.734–2.386)		1.312(1.110–1.551)	
**Size (cm)**				
≤5	1	0.003	1	0.863
>5	1.183(1.058–1.324)		1.010(0.897–1.138)	
**Tumor number**				
Solitary	1	<0.001	1	<0.001
Multiple	1.237(1.101–1.388)		1.246(1.107–1.403)	
**Location**				
Cecum	1	0.779		
Ascending	1.012(0.892–1.148)			
Hepatic Flexure	0.903(0.726–1.123)			
Transverse Colon	1.014(0.862–1.193)			
**Differentiation**				
Grade I/Grade II	1	<0.001	1	0.011
Grade III/Grade IV	1.492(1.323–1.683)		1.175(1.038–1.330)	
**Stage(TNM 7ed)**				
I	1	<0.001	1	<0.001
II	1.127(0.890–1.428)		1.091(0.855–1.392)	
III	1.981(1.573–2.495)		2.872(2.241–3.681)	
IV	6.599(5.214–8.352)		9.854(7.526–12.902)	
**Surgery type**				
PC	1	0.599		
RHC	1.039(0.902–1.197)			
**Postoperative chemotherapy**				
No/Unknown	1	0.073	1	<0.001
Yes	1.108(0.990–1.240)		0.480(0.419–0.551)	

**Table 4:** Univariate and multivariable analysis of factors associated with cancer-specific survival of MAC of RCC.

Variable	Univariate		Multivariate	
	RR(95%CI)	*P*	RR(95%CI)	*P*
**Race**				
White	1	0.201		
Black	1.154(0.935–1.425)			
Others	0.846(0.624–1.148)			
**Age (years)**				
≤ 60	1	0.010	1	<0.001
>60	1.244(1.053–1.470)		1.603(1.342–1.916)	
**Sex**				
Female	1	0.415		
Male	0.944(0.823–1.084)			
**CEA**				
Normal	1	<0.001	1	<0.001
Elevated	2.291(1.986–2.643)		1.428(1.225–1.664)	
**PNI**				
Absent	1	<0.001	1	<0.001
Present	2.864(2.404–3.412)		1.436(1.196–1.726)	
**Size (cm)**				
≤5	1	<0.001	1	0.076
>5	1.428(1.239–1.645)		1.143(0.986–1.326)	
**Tumor number**				
Solitary	1	0.454		
Multiple	0.944(0.812–1.098)			
**Location**				
Cecum	1	0.269		
Ascending	0.875(0.747–1.025)			
Hepatic Flexure	0.844(0.644–1.107)			
Transverse Colon	0.999(0.820–1.217)			
**Differentiation**				
Grade I/Grade II	1	<0.001	1	0.001
Grade III/Grade IV	1.870(1.620–2.158)		1.288(1.112–1.493)	
**Stage(TNM 7ed)**				
I	1	<0.001	1	<0.001
II	2.239(1.331–3.765)		2.002(1.183–3.385)	
III	7.720(4.674–12.751)		9.293(5.553–15.554)	
IV	31.162(18.860–51.489)		36.099(21.298–61.187)	
**Surgery type**				
PC	1	0.407		
RHC	1.078(0.903–1.287)			
**Postoperative chemotherapy**				
No/Unknown	1	<0.001	1	<0.001
Yes	0.534(0.466–0.612)		0.571(0.488–0.669)	

The analyses for CSS showed age, CEA level, PNI, tumor size, differentiation, TNM stage, and postoperative chemotherapy were significant prognostic factors in univariate analyses (P < 0.05, respectively); the multivariable analyses excluded tumor size (P = 0.076). The tumor location and surgery type were neither significant for CSS (both P > 0.05, **Table 4**).

These analyses indicated age, CEA level, PNI, differentiation, and TNM stage were independent prognostic risk factors for both OS and CSS of RCC with MAC. However, RHC surgery type was not a superior prognostic risk factor for OS and CSS compared with PC.

### Long-Term Survival of of Right-Sided Colon Cancer With Mucinous Adenocarcinoma According to Surgery Type

**Figure 3:**
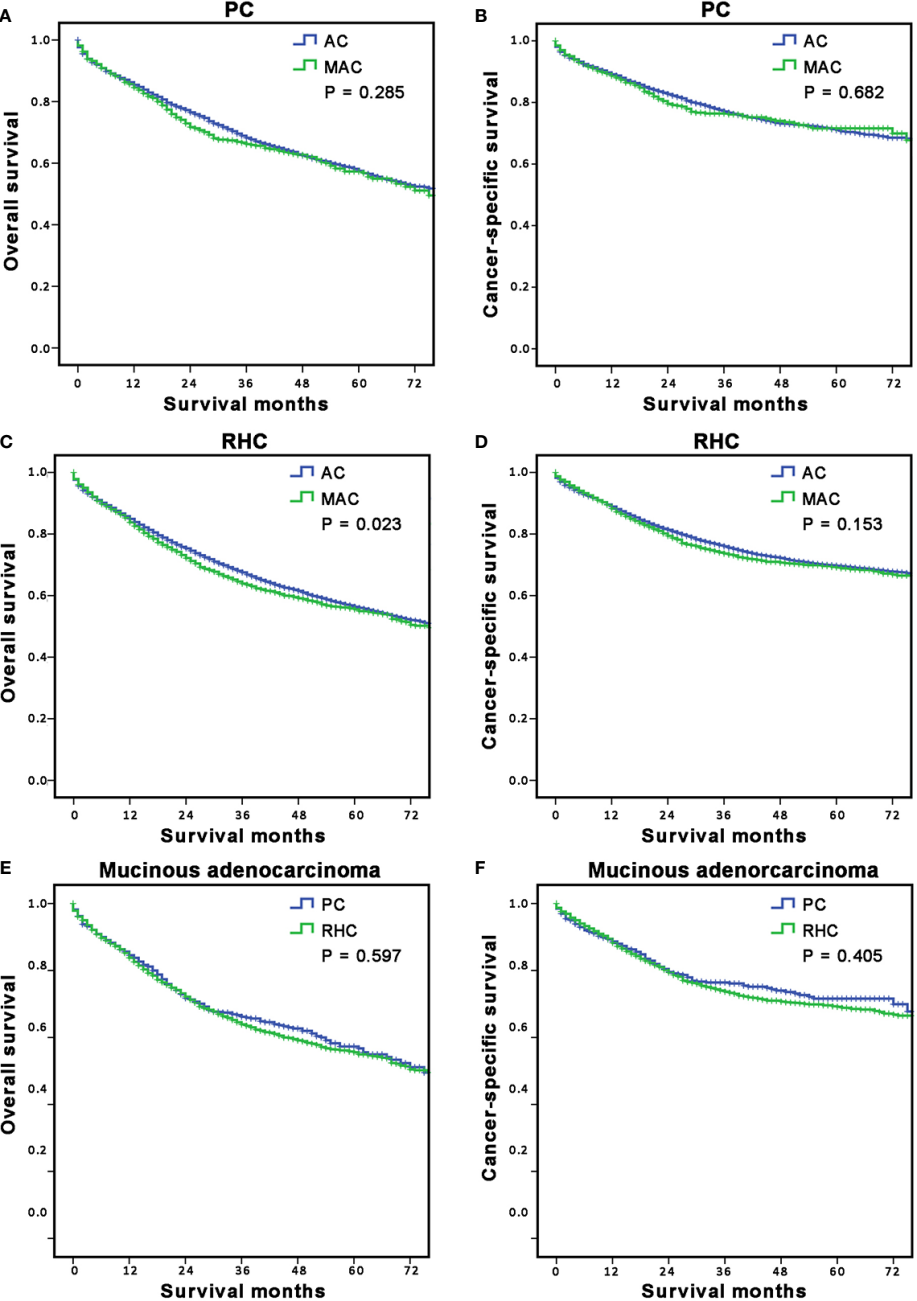
Long-term survival of MAC of RCC according to surgery options. **(A, B)** The stratified analysis survival curves showed that the MAC of RCC group who underwent PC had comparable OS **(A)** and CSS **(B)** with the AC of RCC group; **(C, D)** The stratified analysis survival curves showed that the MAC of RCC group who underwent the RHC had worse OS **(C)** but similar CSS **(D)** with the AC of RCC group. **(E, F)** The survival curves showed that the MAC of RCC patients in the RHC group had similar OS **(E)** and CSS **(F)** as the PC group. PC, partial colectomy; RHC, right hemicolectomy.

After we concluded the different characteristics and risk factors of RCC with MAC, we intended to explore the long-term survival of the MAC group and the subgroup based on surgery type. First, the OS and CSS of all MAC and AC of RCC patients were analyzed according to surgery type (**Figures 3A–D**). The results showed that OS (P = 0.285, **Figure 3A**) and CSS (P = 0.682, **Figure 3B**) of the MAC group were both comparable with those of the AC group when stratified by PC. However, the OS of the MAC group in RHC sub-hierarchy was worse than that of the AC group (P = 0.023, **Figure 3C**), but the CSS was similar between the two group (P = 0.153, **Figure 3D**).

Then, the OS and CSS of RCC with MAC patients were analyzed according to surgery type. The results showed that there was no significant difference in OS (P = 0.597, **Figure 3E**) and CSS (P = 0.405, **Figure 3F**) between the PC and RHC groups. These results indicated that histological subtype was the decisive factor for OS of RCC, especially in RHC sub-hierarchy.

### Additional Sub-Hierarchy Analyses for Long-Term Survival of Right-Sided Colon Cancer

**Figure 4:**
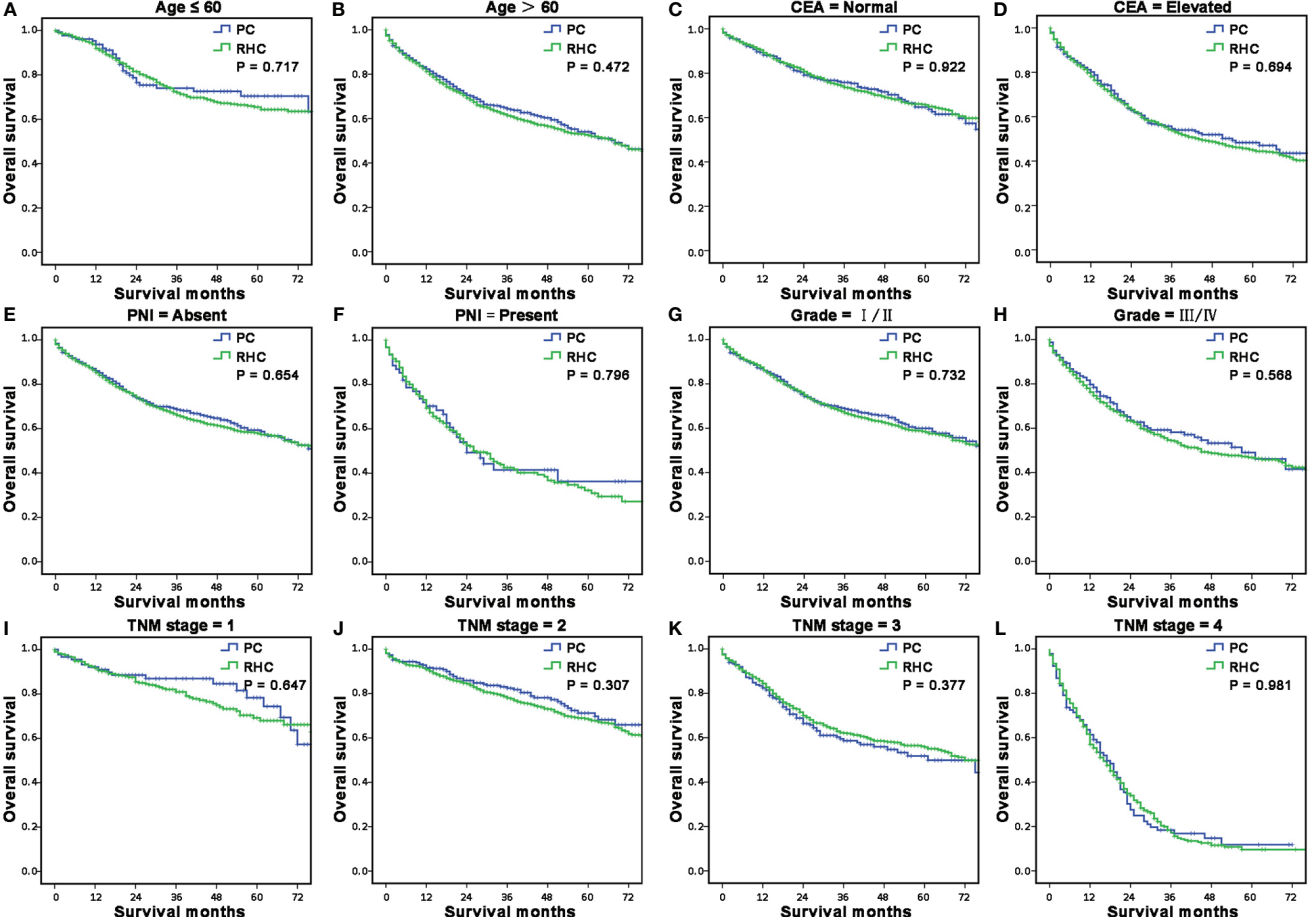
The sub-hierarchy analyses for long-term survival of MAC of RCC according to independent risk factors. **(A, B)** The survival curves showed that the MAC of RCC patients in the RHC group had similar OS and CSS as the PC group when sub-stratified by age **(A, B)**, CEA level **(C, D)**, PNI **(E, F)**, differentiation **(G, H)** and TNM stage **(I–L)**.

Because the multivariable analyses indicated age, CEA level, PNI, differentiation, and TNM stage were independent prognostic risk factors for both OS and CSS of RCC with MAC. Then we further compared the survival between PC and RHC groups stratified by these risk factors. Results showed that the PC group had a similar OS (P > 0.05, respectively, **Figures 4A–L**)and CSS (P > 0.05, respectively, **Supplementary Figures A–L**) with RHC group, no matter which risk factors were sub-hierarchically analyzed.

## Discussion

Surgical resection plays a fundamental role in treating RCC, of which PC and RHC are the most common options (5). However, surgical decision-making for RCC is still controversial, especially in the range of bowel resection (16). Today, many surgeons tend to select RHC for many reasons; the predominant causes are oncology concerns but ignorance of bowel preservation. Moreover, the surgical decision is not specified clearly enough in existing guidelines, especially in the context of histopathology classification. MAC is a specific but not rare histopathological subtype of CRC that has unique demographic and clinicopathological features and potential poor survival according to previous studies as well as this study (17). However, there are still no treatment guidelines or published studies to guide the management of MAC of RCC (6, 10).

Although MAC of RCC has relatively poor survival, surgery is still the key treatment method, but the selection strategy of surgery type is rarely known (10, 18). Interestingly, we found MAC of RCC accepted RHC always had younger age, larger tumor size and cecum or ascending colon location in this study, which indicated a selection tendency of decreasing operating difficulty and increasing patient safety for surgeons but not based on oncology status. In other words, these differences came from the inherent characteristics of surgery types, but didn’t reflect the survival advantage. These findings were also supported by some former studies (19, 20). These findings suggested more evidence-based medicine studies were needed for surgery selection guidance in MAC of RCC.

According to this study, we also concluded that patients could obtain similar OS and CSS from PC or RHC in MAC of RCC. This finding was consistent with a few previous studies with relatively small sample size, in which extensive operation would not be beneficial for CRC patients, especially in RCC (19, 21). We further confirmed that PC could achieve similar long-term survival as RHC, regardless of stratification analysis of the demographic and clinicopathological factors in RCC of MAC. This conclusion overturned the traditional concept that RHC would provide better survival benefits than PC for RCC and also provided the first evidence that PC would be a non-inferiority selection for MAC of RCC. There are some possible explanations for this in the following. First, PC could provide an effective and appropriate extent of colectomy which is consistent with the criteria in the existing guidelines, so extensive colectomy, such as RHC, maybe unnecessary (22). Second, some surgeons suggest the purpose of RHC is to dissect more lymph nodes, but several studies have found that MAC patients had a lower lymph nodes’ metastatic rate than AC patients, thus RHC might not be necessary (23). Third, previous studies suggested that PC had the advantages of a smaller resection range and lower operation stress than RHC, which could potentially provide survival benefit (24). These possible reasons indicated that surgeons have to make the appropriate choice in surgical option decision-making and should not be too eager to perform the extensive operation.

In fact, the extent of positive lymph node dissection is the key to obtaining favorable long-term survival in CRC (2, 8–11, 25). Some research claimed that RHC could dissect more lymph nodes than PC (26, 27). This study showed that the number of dissected lymph nodes in RHC was indeed more than that in PC, but the dissected positive lymph nodes were comparable in the PC and RHC groups. There are also several small sample studies that came to similar conclusions (19, 28). The probable causes of the similar rate of positive lymph node dissection in PC and RHC were as follows: first, the present surgical technique allows most patients to be dissected with sufficient lymph nodes, even though patients undergo relatively less bowel resection range. Second, the lymph node metastases are mainly located in the D1 and D2 groups, which are always along the mesentery and no more than 5 to 10 cm from the primary tumor (29). In addition, D3 lymph node dissection mainly focuses on the central vascular ligation and central lymph node dissection, but does not involve excess resection of the bowel (30). These results further suggest that PC is still an alternative treatment for positive lymph node dissection in patients with MAC of RCC.

There are also several limitations to the present study. First, this is a retrospective study of public databases, which limits the data source due to a lack of homogeneity. Second, due to limitations of the SEER database, we were unable to assess some information such as vascular-lymphatic invasion, postoperative complications, as well as hospital stay time; with these data we could obtain more information, for instance, the operation stress. Lacking support of large multicenter prospective randomized controlled trials is another weakness of the research.

In conclusion, this large population-based study provides a new perspective in the treatment of patients with MAC of RCC and finds that RHC could not dissect more positive lymph nodes, or provide any long-term survival benefit. Moreover, this study could provide some evidence for an update of guidelines for MAC of RCC, which have important clinical value in individual management of colon carcinoma patients.

## Data Availability Statement

Publicly available datasets were analyzed in this study. These data can be found here: Surveillance, Epidemiology, and End Results (SEER) database (https://seer.cancer.gov/).

## Ethics Statement

Institutional review board approval and written informed consent are not required for publicly available data, and none of the potentially identifiable images or data is used in this article.

## Author Contributions

JH, KF, XP, and SX conceptualized the study, conducted the formal analysis, acquired funding, provided the software, and wrote, reviewed, and edited the manuscript. JH, QH, RT, GC, YZ, RH, XZ, KF, and SX performed the data curation, methodology, project administration. SX, KF, and XP: the manuscript revision. All authors contributed to the article and approved the submitted version.

## Funding

This work was supported by the National Natural Sciences Foundation of China (81702949, 31900561), the Natural Sciences Foundation of Hunan Province (2018JJ3851), and the Scientific Research Fund Project of Hunan Provincial Health Commission (20201919, 2017013).

## Conflict of Interest

The authors declare that the research was conducted in the absence of any commercial or financial relationships that could be construed as a potential conflict of interest.
