# Supplementary figures and images for: Hemicolectomy Does Not Provide Survival Benefit for Right-Sided Mucinous Colon Adenocarcinoma
Source: Front Oncol. 2021 Feb 1;10:608836. doi: 10.3389/fonc.2020.608836 (PMC7882730; doi:10.3389/fonc.2020.608836)

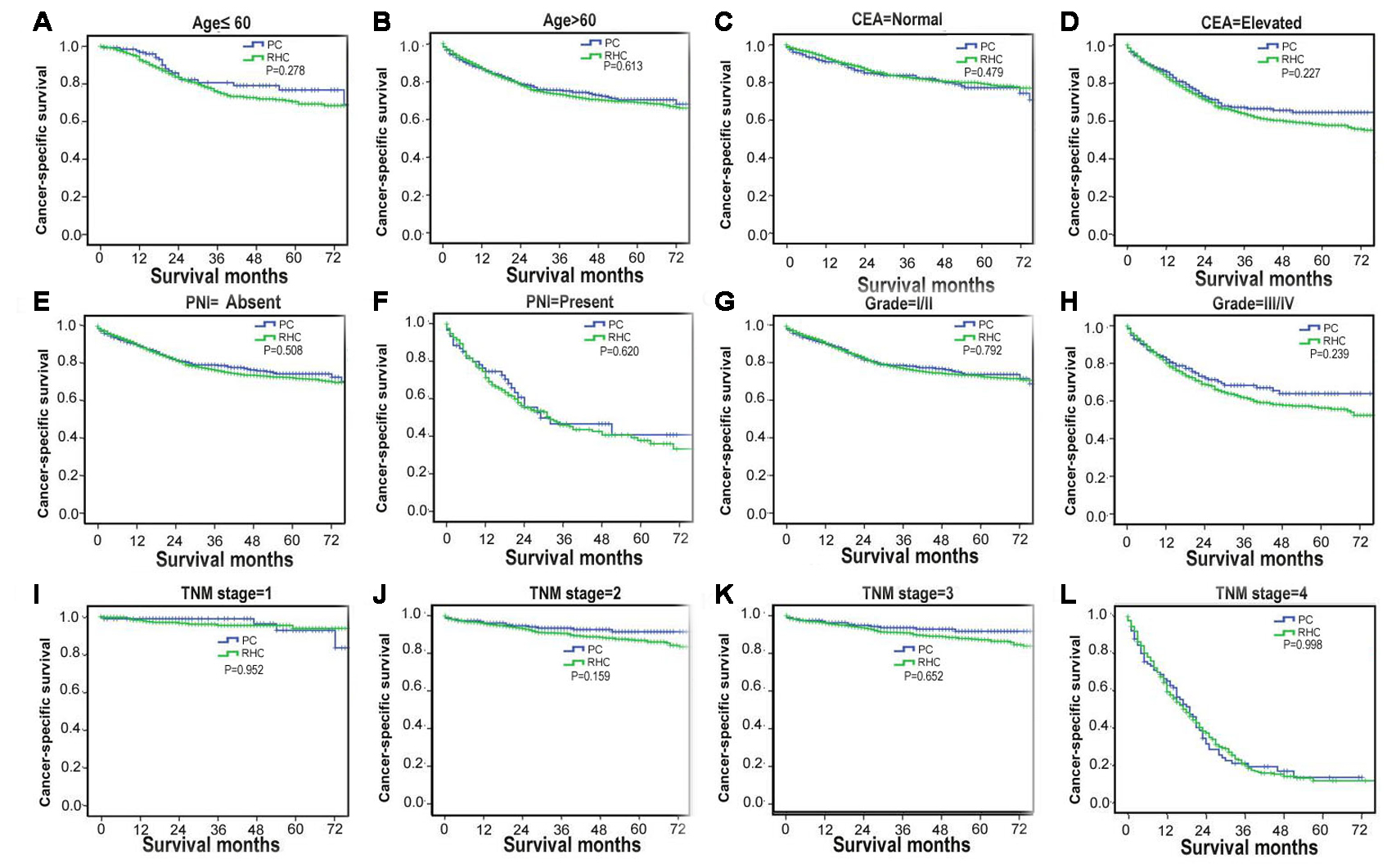

Supplement: Supplementary file 1 [file Image_1.jpeg]
